# The Progress of Mental Treatment in Scotland

**Published:** 1914-07-25

**Authors:** 


### THE PROGRESS OF MENTAL TREATMENT IN SCOTLAND.

### Boarding Out or Institutional Treatment?

The recently-issued Report of the Commissioners in Lunacy for Scotland* may be said to mark an epoch in the history of lunacy legislation and supervision in that country: it is the last Report which will emanate from the Board of Commissioners, as a new *régime* has been initiated by the coming into force of the Mental Deficiency and Lunacy (Scotland) Act, 1913. The Board was instituted in 1857, and since that date many reforms have been brought about in regard to the housing and supervision of the insane. The beneficent teaching of Pinel and of Tuke was slowly permeating the minds of those whose duty it was to make provision for the treatment and care of the insane: Gardiner, Hill, and Conolly were carrying on the good work despite much hostile criticism from the obstinately conservative school which opposed what they designated scornfully as Utopian ideals.

Much, however, remained still to be done; in an admirable summary of "Lunacy Administration since 1857," which is embodied in this Report it is stated that when the Board came into office they were "confronted with the problem of insufficient asylum accommodation to meet the requirements of the insane under their jurisdiction, and also with the fact that a large proportion of the patients in private dwellings throughout the country were living under unfavourable conditions." Their first endeavour, therefore, was to improve the circumstances of the insane in private dwellings. It was from their action in regard to these patients that the system known as "boarding-out" arose; a method of dealing with large numbers of the insane still made use of in Scotland, and even at the present time found to be efficacious and satisfactory. A quite considerable amount of space is given up in the Report to this subject of the care of the insane in private dwellings, and that it is a very important and anxious portion of the work of the Commissioners will be realised when the numbers of the patients in institutional care and of those residing in private houses are considered. Whilst there are just over sixteen thousand in mental hospitals, there are over three thousand in private homes. "The guarantee for the proper care and treatment of 'pauper lunatics' in private dwellings is one of the most important matters connected with this branch of lunacy administration in Scotland. Its essential feature is the obligation placed upon every inspector of poor to give intimation to the Board of every such person in his parish as soon as such case becomes known to him." It is not necessary that these patients should be sent to an asylum; the Board may sanction their remaining in private care if they are satisfied that adequate provision is being made for their care and treatment.

* *The Fifty-sixth Report of the General Board of Commissioners in Lunacy for Scotland.* 1914.

Visitation by the inspector of poor must be made at least twice a year, and by a Commissioner at least once. "The Board has," the Report states, "a very complete knowledge of the condition and history of every pauper lunatic in private dwellings, and in practice the guarantees for proper treatment are found to be ample." The maintenance rate of these boarded-out patients averages about 7s. a week, or with clothing and medical attendance about 8s. 6d. a week; that is to say, the expenditure is "considerably below the average cost of maintenance in asylums." The conditions in regard to health are favourable: the death rate in the quinquenniad 1908-1912 was but 3.8 per cent. Only two serious cases of assault have been recorded during a space of fifty-six years among an insane population averaging in number between 2,000 and 3,000 persons. "In considering the growth of this system, it is surprising to observe that, originating as it did in the exigencies of the crude and rudimentary methods of caring for the insane which existed in Scotland prior to 1857, it has yet developed under very slender legislative sanction into a permanent and established system which could not without enormous expense to the country be superseded." The Commissioners, with their prolonged experience of this system of "boarding out," are satisfied that "the great majority of those patients who have had experience of life in asylums and of life in private dwellings greatly prefer the more social and unrestricted life which they enjoy under the system of private care." It must be remembered in considering this aspect of the question of dealing with the insane that very adequate supervision is provided for: it is an essential part of the system, for without it abuses in the shape of neglect or even of cruelty would almost certainly exist. Human nature has not radically changed since the Royal Commission of 1855 wrote that "although the inhabitants of the districts may generally show kindness to these unhappy persons, they doubtless are exposed to much suffering and privation, and occasionally end their days in a condition inconceivably wretched."

Another essential matter with which this Report deals is that of the recognition of the importance of hospital accommodation in asylums. The days are happily past when the routine treatment of the acutely insane was to insist upon their being kept constantly on the move in order that they might in this way the sooner get rid of what was regarded as superfluous energy. It is now recognised that this energy must be carefully conserved instead of being recklessly dissipated; and, just as in other acute bodily disorders rest is the chief desideratum, so in acute mental (or brain) states bed-treatment is a prime necessity. In debilitated and in senile cases it almost goes without saying that repose is equally necessary. So it has been found that whereas "thirty years ago it was considered sufficient that the hospital section should afford accommodation for about one-third of the total number of inmates," nowadays "one-half of the whole accommodation is required for hospital purposes."

There are many other important matters dealt with in this Report—e.g., "The Extension of Asylum Lands," "The Abolition of Airing Courts," "The Nursing of Male Patients by Female Nurses." Considerations of space, however, preclude the discussion of these in the meantime. Enough has, however, been said to indicate the enlightened view which is taken by those appointed to supervise the insane in Scotland.

